# The *Drosophila* Split Gal4 System for Neural Circuit Mapping

**DOI:** 10.3389/fncir.2020.603397

**Published:** 2020-11-09

**Authors:** Haojiang Luan, Fengqiu Diao, Robert L. Scott, Benjamin H. White

**Affiliations:** Laboratory of Molecular Biology, National Institute of Mental Health, NIH, Bethesda, MD, United States

**Keywords:** intersectional targeting, binary expression systems, cell type, connectomics, genetic models

## Abstract

The diversity and dense interconnectivity of cells in the nervous system present a huge challenge to understanding how brains work. Recent progress toward such understanding, however, has been fuelled by the development of techniques for selectively monitoring and manipulating the function of distinct cell types—and even individual neurons—in the brains of living animals. These sophisticated techniques are fundamentally genetic and have found their greatest application in genetic model organisms, such as the fruit fly *Drosophila melanogaster*. *Drosophila* combines genetic tractability with a compact, but cell-type rich, nervous system and has been the incubator for a variety of methods of neuronal targeting. One such method, called Split Gal4, is playing an increasingly important role in mapping neural circuits in the fly. In conjunction with functional perturbations and behavioral screens, Split Gal4 has been used to characterize circuits governing such activities as grooming, aggression, and mating. It has also been leveraged to comprehensively map and functionally characterize cells composing important brain regions, such as the central complex, lateral horn, and the mushroom body—the latter being the insect seat of learning and memory. With connectomics data emerging for both the larval and adult brains of *Drosophila*, Split Gal4 is also poised to play an important role in characterizing neurons of interest based on their connectivity. We summarize the history and current state of the Split Gal4 method and indicate promising areas for further development or future application.

## Introduction

At the end of his scientific autobiography, Francis Crick presciently noted that for neuroscience research to progress “it would be useful to be able to inactivate, preferably reversibly, a single type of neuron in a single area of the brain” (Crick, 1988). This desideratum was motivated by the crudeness of available methods for manipulating brain activity. When this passage was written, the technologies that would enable more refined neural manipulations were already being created, as molecular biology—Crick’s first field of endeavor—steadily revolutionized other areas of biology. In 1982, Rubin and Spradling (1982) had demonstrated that a eukaryotic transposon could be used to ferry a foreign gene into the germline of a metazoan—*Drosophila*—and be expressed in its somatic cells. Using this method of germline transformation, Mark Ptashne’s group demonstrated in 1988—the same year Crick’s autobiography was published—that the yeast transcription factor, Gal4, could drive the expression of a second transgene introduced into the fly genome behind Gal4’s DNA recognition site, or Upstream Activating Sequence (UAS, Fischer et al., 1988). Within a scant 5 years, Brand and Perrimon (1993) generalized this capability, creating a method for yoking Gal4 expression to the regulatory elements of randomly targeted genes, and within another 2 years, this “Gal4-UAS system” had been used to direct the expression of a neuronal suppressor, tetanus toxin light chain, to specific subsets of neurons in the fly brain (Sweeney et al., 1995). Reversible inactivation became possible in 2002 with the creation of a UAS-expressible version of Shi^ts1^, a temperature-sensitive, dominant-negative mutant of the *Drosophila* dynamin gene which is required for sustained neurotransmission (Kitamoto, 2002). Methods for temperature-mediated neuronal activation followed, as did the explosive development of “optogenetic” tools for light-mediated neuronal activation and inactivation (Bernstein et al., 2012). Today, the toolkit of effector transgenes available to neurobiologists to manipulate and monitor neuronal function in flies and other genetic model organisms make Crick’s original request seem somewhat quaint (Hampel and Seeds, 2017; Martin and Alcorta, 2017; Luo et al., 2018). Although it remains an aspirational goal to be able to selectively target the expression of such transgenes to each individual neuronal cell type in an animal, advances in genetic targeting techniques are placing even this goal within reach.

**Figure 1:**
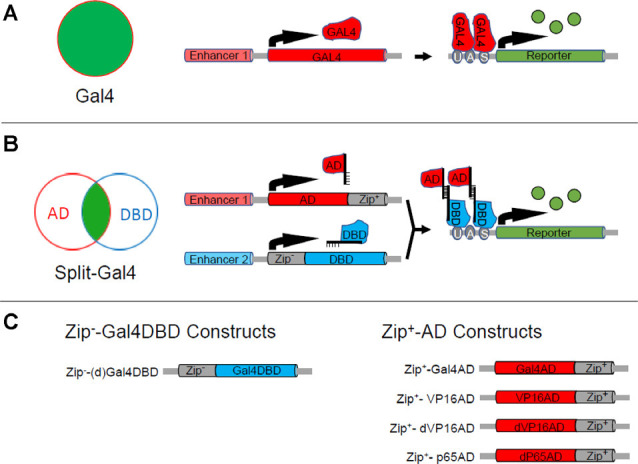
The Split Gal4 system. **(A)** The binary Gal4-UAS expression system can be used to target the expression of a reporter or effector (green) to a group of cells (red circle) in which an enhancer (Enhancer 1) is active. As illustrated in the right-hand schematics, Enhancer 1 drives expression of the Gal4 transcription factor (red) which in turn drives expression of the reporter or effector gene (green), which is placed downstream of Gal4’s DNA binding site (UAS). **(B)** The Split Gal4 system uses two enhancers with activity in overlapping cell groups (red and blue circles) to target reporter or effector expression (green) to the intersection of the two groups. The intersectional logic of expression is shown schematically in the Venn diagram (left). The right-hand panels illustrate the transcriptional mechanisms: one enhancer (Enhancer 1) is used to target expression of the transcription activation domain (AD) of Gal4 or some other transcription factor fused to the Zip^+^ leucine zipper, while the other (Enhancer 2) is used to target expression of the Gal4 DNA binding domain (Gal4DBD) fused to the Zip^+^ leucine zipper. Association of Zip^+^ and Zip^+^ brings the GalDBD and AD components together to reconstitute Gal4 transcriptional activity and drive expression of UAS-transgenes. **(C)** Design of Zip^−^-Gal4DBD and Zip^+^-AD constructs. Two Zip^−^-Gal4DBD constructs have been made. They share the same sequence, but the one made by Pfeiffer et al. (2010; designated here as dGal4DBD) is codon-optimized for use in *Drosophila* and is placed behind a *Drosophila* synthetic core promoter. Activation domains from three different transcription factors have been used to make Zip^+^-AD constructs. Zip^+^-p65AD and Zip^+^-dVP16AD are codon-optimized and show strong, high-fidelity expression.

Cell types are fundamentally distinguished by the genes that they express and genetic methods for targeting particular cell types follow a common strategy. The genetic regulatory elements (i.e., enhancers) of cell-type-specific genes are conscripted to drive the expression of an activator, such as Gal4. Just as in the two original implementations of the Gal4-UAS system, this can be done in two ways. A Gal4 construct can be fused to identified enhancer fragments of a native gene so that Gal4 is expressed under the control of these enhancers when the construct is inserted into the genome (Figure 1A). Alternatively, a Gal4 expression construct can be inserted into or near a gene in such a way that Gal4’s expression is driven by the endogenous enhancers regulating the expression of that gene. Because few genes—and more specifically, few enhancers—are truly cell-type specific, this strategy usually must be augmented by other methods for further delimiting either Gal4 expression or—what has been more generally useful—its scope of activity.

Gal4’s transcriptional activity can be directly blocked by an extremely effective natural inhibitor encoded by the yeast gene Gal80. By placing Gal80 expression under the control of a second enhancer, the activity of which overlaps with that of the enhancer(s) driving Gal4 activity, one can restrict Gal4 activity to only cells in the non-overlapping part of the expression pattern (Lee and Luo, 1999; McGuire et al., 2004). This strategy is often described as implementing a logical NOT gate on Gal4 expression. While strategies that effect NOT gates are useful in excluding cells or cell types from a Gal4 expression pattern, methods that permit positive, rather than negative, selection have distinct advantages in selecting cell types. Positive selection, by implementing a logical AND function, allows one to isolate cell types based on two genes that they co-express rather than one which they co-express and one that they do not. One combinatorial strategy for implementing an AND gate impairs not Gal4 activity *per se*, but instead its ability to activate the expression of a particular UAS-transgene. This is accomplished by interposing a recombinase-removable translational “stop cassette” between the UAS sequence and the sequence encoding the transgene (Stockinger et al., 2005). Removal of this cassette in cell types that express the recombinase effectively restricts the scope of Gal4 activity to only those cell types that express the recombinase. A disadvantage of this strategy is that it requires a unique recombinase-sensitive version of each UAS-transgene that one might want to express. Also, the excision of the stop cassette is permanent, which can result in transgene expression in unwanted cell types if there is developmental variation in the pattern of recombinase expression. A more general strategy that permits positive selection is a derivative of the Gal4-UAS system called Split Gal4.

Like the Gal4-UAS system, the development of Split Gal4 was facilitated by insights derived from the earlier molecular biological investigation of transcription factor properties. Gal4 had been shown to possess distinct protein domains for binding to DNA and for activating transcription (Keegan et al., 1986; Ma and Ptashne, 1987). These two domains were incapable of promoting gene expression alone when separated, but if fused to interaction domains that brought them together they could reconstitute Gal4 transcriptional activity. This capacity became the basis of the “yeast two-hybrid” system widely used to identify naturally occurring protein-protein interaction domains (Fields and Song, 1989). By fusing the DNA-binding (DBD) and transcription activation (AD) domains of Gal4 to strong, heterodimerizing leucine zippers, Luan et al. (2006) exploited this feature of Gal4 to create a system in which the two Gal4 domains could be independently targeted to different cells using distinct enhancers (Figure 1B). Only those cells in which both enhancers were active would express both Gal4 components and thus reconstitute Gal4 activity. This Split Gal4 method can target single cells or cell types in the *Drosophila* nervous system where it has found its greatest application. With a range of tools now available to facilitate its application, it has become the workhorse for mapping neural circuits in the fly. This method—its development, its essential toolbox, its application, and its potential for future use—is the subject of this review.

## The Split Gal4 Toolkit

### The Parts List

#### Original Instruments

When the Split-Gal4 system was introduced, it consisted of three components: the Gal4 DNA binding domain (Gal4DBD; amino acids 1–147 of the native Gal4 sequence) and two alternative transcription activation domains (AD; Figure 1C). The first AD corresponded to the native Gal4AD (i.e., “Gal4AD II,” amino acids 768–881), while the second corresponded to the more potent AD domain of the herpes simplex virus transcription factor VP16. The Gal4DBD was fused *via* a short linker to one of a pair of high-affinity, heterodimerizing leucine zippers (Moll et al., 2001, called here for simplicity Zip^+^ and Zip^−^), while the Gal4AD and VP16AD were fused to the complementary zipper to cause them to associate with the Gal4DBD when both components were expressed in the same cell. Fly lines individually expressing the Zip^−^-Gal4DBD and Zip^+^-AD constructs were termed “hemidrivers,” and pairing the Zip^−^-Gal4DBD with either a Zip^+^-Gal4AD or Zip^+^-VP16AD hemidriver was shown to promote transcription of UAS-transgenes. The Zip^−^-Gal4DBD/Zip^+^-VP16AD pair had the advantage of being considerably more efficacious in doing so.

**Figure 2:**
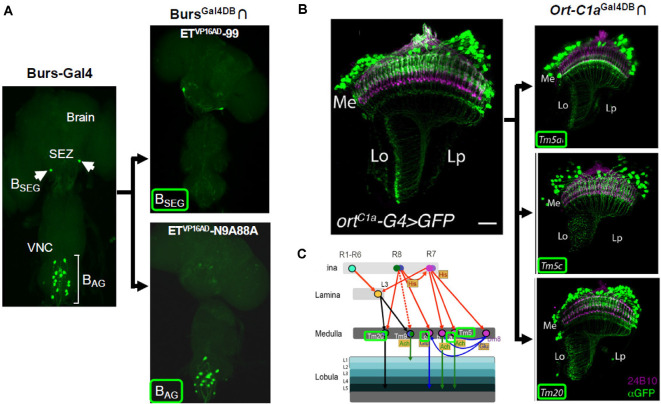
Cell type-specific expression achieved with Split Gal4. **(A)** The B_AG_ and B_SEG_ are groups of neurons in the ventral nerve cord (VNC) and subesophageal zone (SEZ), respectively, that express the hormone, Bursicon (left). A Burs^Gal4DBD^ hemidriver in combination with two distinct enhancer-trap Zip^+^-VP16AD hemidrivers can be used to individually target each group (right panels). **(B,C)** Split Gal4 parsing of medulla neurons. **(B)** Medulla neurons that receive input from photoreceptors are labeled by the *ort-C1a*-Gal4 driver (ort^C1a^-G4, left). Three subpopulations of these neurons, Tm5a, Tm5c, and Tm20, with different projection patterns, are identified by an *ort-C1a^Gal4DBD^* hemidriver used with different enhancer-trap Zip^+^-dVP16AD hemidrivers (right panels). **(C)** Schematic showing input and output patterns of Tm5a, Tm5c, and Tm20 neurons (names outlined in green). Panel **(A)** adapted from Luan et al. (2012); panels **(B,C)** from Melnattur et al. (2014).

A downside of the Zip^+^-VP16AD construct, however, was that when expressed under the control of specific enhancers it showed significant ectopic expression and was not therefore useful for precise targeting. Luan et al. (2006) demonstrated that the Zip^−^-Gal4DBD could be faithfully expressed in specific populations of cells using defined enhancers and used with enhancer-trap Zip^+^-VP16AD lines to restrict expression to smaller groups of cells within the population. They subsequently demonstrated the efficacy of this approach using a Zip^−^-Gal4DBD driven under the control of the promoter for Bursicon, a hormone that is critical for the expansion and hardening of the wings after the emergence of adult flies (Luan et al., 2012). By screening a library of several hundred Zip^+^-VP16AD enhancer-trap lines, the authors isolated Split Gal4 hemidriver pairs that selectively expressed in Bursicon-expressing neurons of either the abdominal or subesophageal ganglia (Figure 2A). They used these lines to demonstrate that activation of a single pair of neurons in the subesophageal zone (SEZ) was sufficient to command wing expansion in newly eclosed flies. Similarly, the Jefferis laboratory generated a much larger Zip^+^-VP16AD hemidriver library of approximately 2,000 lines, which they screened to isolate subsets of cholinergic neurons that expressed the transcription factor fruitless (Kohl et al., 2013) and subsets of neurons with expression in the lateral horn (Frechter et al., 2019).

#### Improved AD Constructs

An alternative version of the VP16AD construct, in which a potential Hox gene binding site had been eliminated and the codon usage had been optimized for expression in *Drosophila*, showed considerably higher fidelity. This AD, called “dVP16AD,” (Figure 1C) was first used to restrict expression of Zip^+^-dVP16AD to a subset of glutamatergic targets of the R7 photoreceptors in a study of the neural circuitry underlying color discrimination in flies by Gao et al. (2008). When paired with a Zip^−^-Gal4DBD expressed under the control of an enhancer for the histamine-gated chloride channel encoded by the *ort* gene (i.e., *ort*^Gal4DBD^), the *vGlut*^dVP16AD^ hemidriver restricted expression to three distinct glutamatergic cell types in the optic lobe, including the Dm8 neurons, which were shown to be responsible for UV preference. These and other Split Gal4 hemidrivers subsequently found use in the dissection of motion detection circuits in the visual system (Joesch et al., 2010; Clark et al., 2011). Enhancer trap production of Zip^+^-dVP16AD lines by Melnattur et al. (2014) was subsequently used to identify hemidriver pairs which in combination with an *ort*^Gal4DBD^ identified specific subsets of first-order projection neurons of the medulla involved in color discrimination (Figures 2B,C).

A second Split Gal4 AD construct made with the activation domain of the human p65 transcription factor (i.e., Zip^+^-p65AD; Figure 1C) was introduced in 2010 by Pfeiffer et al. (2010) who demonstrated that this construct, like dVP16AD, could drive robust and high-fidelity expression of a reporter transgene in combination with a Zip^−^-Gal4DBD. The Zip^+^-p65AD, together with a *Drosophila* codon-optimized version of the Zip^−^-Gal4DBD (i.e., dGal4DBD; Figure 1C) introduced by the same authors has seen subsequent widespread use. Numerous studies have now confirmed the efficacy of the Gal4DBD, dGal4DBD, dVP16AD, and p65AD constructs shown in Figure 1C in a variety of contexts. All can be used to drive expression restricted to the cell-types dictated by the enhancers used to express them. It should be noted that while the fidelity is good, even hemidrivers made with the optimized Split Gal4 constructs can exhibit occasional expression that is not evident in the patterns of parent Gal4 drivers, and verification of fidelity may be necessary in critical cases (Pfeiffer et al., 2010; Cichewicz et al., 2017).

#### A Split Gal4 Repressor: the Killer Zipper

**Figure 3:**
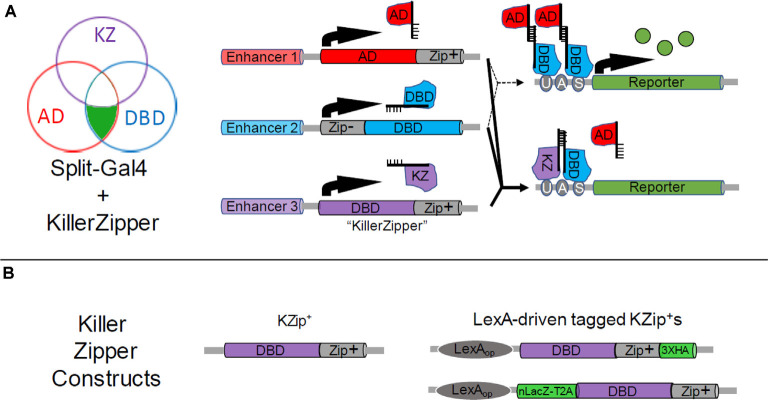
Restricting Split Gal4 expression using the Split Gal4 repressor, KZip^+^. **(A)** As shown in the Venn diagram (left), the Killer Zipper (KZip^+^) can be used to exclude Split Gal4 activity from cells within an intersection. Where the activity of the enhancer used to drive KZip^+^ expression (Enhancer 3, purple circle) overlaps with that of the enhancers used to drive expression of the Zip^+^-AD (red circle) and Zip^−^-Gal4DBD (blue circle) constructs (see Figure 1B), Split Gal4 activity is repressed and no reporter expression is observed. This is illustrated in bottom-right panels, where the solid lines indicate the expression of all three constructs. Reporter expression is restricted to only that part of the intersection where Zip^+^-AD and Zip^−^ -Gal4DBD alone are expressed (upper right-hand panels; dotted lines). **(B)** Available Killer Zipper constructs include a basic KZip^+^ that can be expressed under the control of a specific enhancer (left) and several “universal” constructs that express KZip^+^ constructs under the control of LexA drivers. Two of the latter are shown (right), one of which bears a hemagglutinin (HA) tag and the other of which co-expressed a nuclear LacZ molecule. HA and nLacZ permit the detection of KZip^+^ expression in cells by immunostaining.

Although Zip^+^-dVP16AD and Zip^+^-p65AD hemidrivers both promote significantly more robust expression of UAS-transgenes than the Zip^+^-Gal4AD in Split Gal4 applications, only the latter construct is repressible by Gal80 and is therefore useful for implementing a second (NOT) intersection if the further restriction of expression is required. Although the single intersection effected by Split Gal4 can often provide an impressive reduction in the number of cell types seen with Gal4, it is not uncommon for a Split Gal4 pattern to retain at least a small number of residual cell types outside of the desired pattern. In this case, further restriction of expression can be advantageous. Efforts to make a Zip^+^-Gal4AD construct by changing either the geometry and linker length of the original construct (Luan et al., 2006) or by using the full-length Gal4 activation domain (Pfeiffer et al., 2010) failed to improve efficacy. Dolan et al. (2017) pursued an alternate strategy of creating a repressor for Split Gal4 activity that could serve an analogous purpose to Gal80. The resulting “Killer Zipper” construct (KZip^+^) consists of a dGal4DBD fused to the Zip^+^ leucine zipper so that it competes with KZip^+^-AD constructs for binding to the normal Zip^−^-Gal4DBD (Figure 3A). Because the active Gal4 transcription factor is a dimer, in which two Gal4DBDs form the DNA-binding pocket, the KZip^+^ construct not only competes with AD constructs to form transcriptionally incompetent Gal4DBD dimers, but these dimers can bind to UAS sites and block binding of transcriptionally competent Zip^−^-Gal4DBD-Zip^+^-AD pairs. Because the efficacy of the KZip^+^ construct will depend on its intracellular concentration, which will depend on the strength of the enhancer used to drive its expression, Dolan et al. (2017) created a set of universal KZip^+^ constructs, placed behind a LexAop promoter. High-level expression of these constructs, some of which express tags that can be used to track expression (Figure 3B), can then be attained by using a LexA driver that expresses in the cell type(s) to be eliminated from a pattern. Both LexAop-KZip^+^ and enhancer-driven KZip^+^ constructs have proven useful in delimiting Split Gal4 expression in neuroblasts (Carreira-Rosario et al., 2018; Seroka and Doe, 2019).

### Tools for Targeting Expression

Just as the components of the Split Gal4 system have improved since its inception, so have the methods required for directing their expression to generate useful intersections of enhancer expression patterns. When the Split Gal4 system was introduced, few characterized enhancers existed that could be used to make Split Gal4 lines with gene-specific expression patterns. Methods for converting existing Gal4 enhancer-trap lines with desirable expression patterns into Split Gal4 lines with equivalent expression were also cumbersome as was the process of making and screening new Split Gal4 enhancer trap lines. In the intervening years, numerous technical developments have facilitated progress in all of these areas and many new resources have been generated that give researchers interested in using Split Gal4 a variety of readily implemented options.

#### Generating Split Gal4 Lines With Gene-Specific Expression

An obvious and important use of the Split Gal4 system is to target cell groups that lie at the intersection of expression of two genes of interest. This application might be used for either “cell discovery” or “cell characterization” depending on whether one is trying to identify neurons that express both genes or to characterize the properties of neurons known to be distinguished by their expression of the two genes. In either case, gene-specific expression of the Zip^−^-Gal4DBD and Zip^+^-AD components is required. When the Split Gal4 technique was introduced such gene-specific expression could be achieved either by using one of the few characterized DNA sequences known to contain the enhancers responsible for the expression of a gene or by tediously converting an enhancer-trap Gal4 line known to exhibit gene-specific expression into a corresponding Split Gal4 line using homologous recombination (see for example Gao et al., 2008). Although the number of enhancer fragments that faithfully replicate the expression of a native gene remains small—and most genes are now known to be under the control of multiple, often spatially dispersed, enhancers—techniques for expressing transgenes in a gene-specific manner have been considerably simplified by new methods that permit one to couple the expression of a transgene to that of a native gene or to easily exchange existing Gal4 transgenes with modules containing Split Gal4 components.

Three principal tools underlie new methods. One is the ΦC31 integrase (Groth et al., 2004), which facilitates the modular genetic exchange of constructs into genomic loci at which an attP integrase recognition site has been introduced (Venken et al., 2006; Bischof et al., 2007). The second tool is the Cas9 nuclease, which permits sequence-specific editing at arbitrary genomic loci using CRISPR-based guide RNAs (Gratz et al., 2013; Jinek et al., 2013). The latter tool permits the introduction of highly specific breakpoints in genomic DNA to facilitate transgene replacement by homologous recombination. A final enabling technology that has permitted researchers to co-opt the regulatory elements governing the expression of a gene of interest is the viral T2A peptide (Diao and White, 2012). Insertion of the sequence encoding T2A into a gene of interest causes two independent polypeptides to be translated, one encoded by the sequence before the T2A C-terminus and one encoded by the sequence following it. By placing transgenes encoding Gal4 or Split Gal4 components downstream of a T2A sequence one can co-express them with a gene of interest without explicit knowledge of that gene’s enhancers.

**Figure 4:**
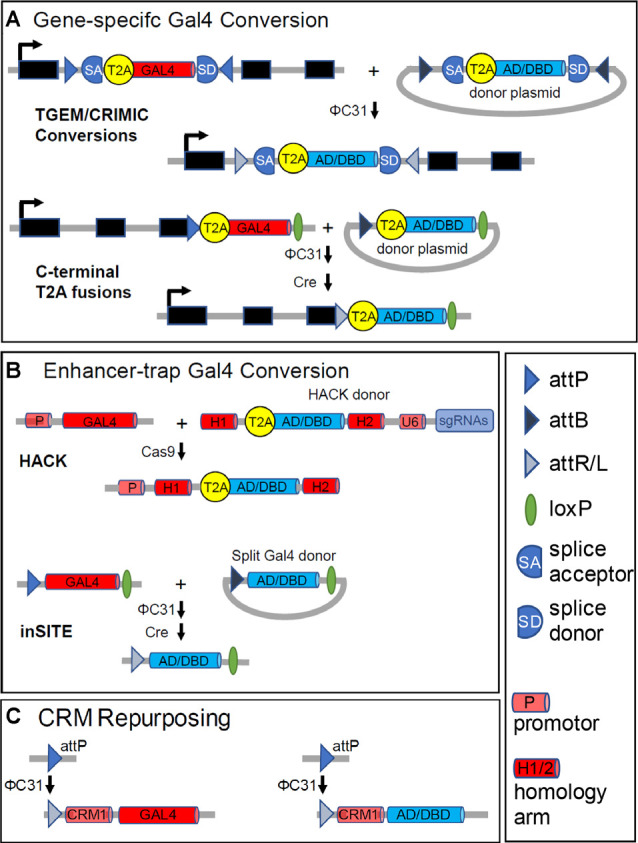
Generating Split Gal4 hemidrivers from Gal4 drivers. While Split Gal4 hemidriver lines can be generated directly, in most instances a Gal4 driver with a desired pattern of expression serves as an intermediate. These starting Gal4 drivers fall into three classes: **(A)** “gene-trap” Gal4 lines with gene-specific expression; **(B)** enhancer-trap Gal4 lines made by transposon-mediated transformation; and **(C)** CRM-Gal4 lines that use enhancer-containing cis-regulatory modules with defined sequences. **(A)** Gene-trap drivers typically couple Gal4 expression to that of a native gene using T2A peptides (see text). T2A-Gal4 constructs are inserted either intronically, as in TGEM or CRIMIC lines (top), or just before the stop codon in the coding sequence (bottom). In both cases, the inclusion of either attP sites or an attP and a loxP site flanking the inserted constructs permits conversion of the Gal4 line into a Split Gal4 hemidriver. A Zip^+^-AD or Zip^−^-Gal4DBD donor construct with complementary flanking attB sites (or an attB and loxP site) can be substituted for Gal4, as indicated. **(B)** Top panels: the CRISPR/Cas-based HACK method (Lin and Potter, 2016; Xie et al., 2018) can be used to convert arbitrary enhancer-trap Gal4 drivers into Split Gal4 hemidrivers using a universal donor construct. The HACK donor construct has a T2A- Zip^+^-AD or -Zip^−^-DNA-BINDING (DBD) sequence flanked by homology arms (H1 and H2) taken from the Gal4 coding sequence. Also, the donor construct has an expression module for guide RNAs targeted to sites in the Gal4 sequence separating H1 and H2. Cas9-mediated cleavage of Gal4 at these sites, followed by homology-directed repair inserts the desired T2A-Split Gal4 construct in-frame into the—now broken—Gal4 sequence. Bottom panels: Enhancer-trap Gal4 lines made using the inSITE system (Gohl et al., 2011) can be converted into Split Gal4 hemidrivers by a series of genetic crosses. The system uses a set of three recombinases (Flp is omitted from the figure for simplicity) to substitute the desired Split Gal4 construct for Gal4 at the site of insertion. **(C)** The sparsely-expressing Gal4 lines made by the Rubin and Dickson labs (Jenett et al., 2012; Kvon et al., 2014) use enhancer fragments with defined sequences (CRMs) to drive Gal4 expression in specific patterns. The CRM-Gal4 constructs are also inserted into defined attP landing sites. A Split Gal4 hemidriver corresponding to a given CRM-Gal4 driver can thus be made by inserting into the identical genomic site (e.g., attP) a construct that uses the same CRM (here “CRM1”) to drive a Zip^+^-p65AD or Zip^−^-Gal4DBD construct instead of Gal4. As Dionne et al. (2018, #114) caution, insertion of the Split Gal4 construct into other genomic sites may lead to deviations from the original expression pattern. The legend indicates symbols used for various DNA motifs.

A technology that makes use of all three tools to permit gene-specific expression of Split Gal4 components is the Trojan exon method (Diao et al., 2015). Trojan exons are synthetic exons that can be introduced into so-called “coding introns” (i.e., introns flanked by exons that contain coding sequence of a gene). The presence of a strong splice acceptor site (SA) before the Trojan exon ensures incorporation of the exon into the mRNA transcribed from the gene into which it is inserted so that its transgene is translated. Using ΦC31, Split Gal4-encoding Trojan exons can readily be inserted into MiMIC transposons located in coding introns (Venken et al., 2011; Figure 4A, top). Approximately 1,500 *Drosophila* genes have such MiMIC transposons and over 600 of these have been converted into Trojan Gal4 lines by the *Drosophila* Gene Disruption Project (GDP) using genetic methods that do not require germline injections (Lee et al., 2018). Germline injections of Split Gal4 constructs are required to generate Split Gal4 lines from the same MiMIC insertions. Many genes do not have MiMIC insertions, but Diao et al. (2015) also created a MiMIC-like “Trojan exon Gal4 expression module” (TGEM) which can be inserted into the coding introns of arbitrary genes using CRISPR/Cas 9 technology. These Gal4 insertions, once in place, can then be easily exchanged for Split Gal4 components using ΦC31. A modified version of the TGEM construct called CRIMIC, which has been designed for easy excision from the genome, is currently being incorporated into several thousand additional *Drosophila* genes by the GDP and will eventually permit Split Gal4 lines to be generated for most genes in the *Drosophila* genome (Lee et al., 2018). The growing number of TGEM and CRIMIC lines, most of which are publicly accessible through the Bloomington *Drosophila* Stock Center (BDSC), represent a valuable resource for making Split Gal4 lines with gene-specific patterns of expression (Table 1).

**Table 1:** Collections of lines for split Gal4 applications.

Line type/specificity	# of lines	References	Useful links
**Collections of split Gal4 convertible Gal4 drivers**		
CRM (GMR Gal4)	~7,000	Jenett et al. (2012)	Janelia FlyLight; FlyLight Gal4; Rubin Lab; FlyLight MCFO
CRM (VT Gal4)	~8,000	Kvon et al. (2014)	VT; Virtual Flybrain; FlyLight GAl4; FlyLight MCFO
Gene trap (Trojan)	~600	Diao et al. (2015); Lee et al. (2018)	GDP MiMIC; BDSC T2A-Gal4
Gene trap (CRIMIC)	~700	Lee et al. (2018)	DGP CRIMIC; BDSC CRIMIC;
Gene trap (Signalling)	~200	Deng et al. (2019); Kondo et al. (2020)
Enhancer trap (InSITE)	~1,200	Gohl et al. (2011)	InSITE Database; BDSC InSITE
**Collections of Split Gal4 hemidrivers**		
CRM (GMR/VT)	~4,000	Dionne et al. (2018); Tirian and Dickson (2017)	BDSC Split Gal4
**Collections of neuroanatomically-specific split Gal4 drivers**		
Lamina	22	Tuthill et al. (2013)	FlyLight Split Gal4
Lobula columnar	22	Wu et al. (2016)	FlyLight Split Gal4
Optic lobe	42	Davis et al. (2020)	FlyLight Split Gal4
Mushroom body	92	Aso et al. (2014a)	FlyLight Split Gal4
Central complex	27	Wolff and Rubin (2018)	FlyLight Split Gal4
Lateral horn	87	Dolan et al. (2019)	FlyLight Split Gal4
Descending neurons	133	Namiki et al. (2018)	FlyLight Split Gal4
Neuroblast-specific	12	Lacin and Truman (2016)	

One particular class of genes of interest to neuroscientists are those that establish the signaling capacities of neurons. The neurotransmitters and neuromodulators to which a neuron is responsive, together with those which it uses to communicate with other cells, are often among its defining features. The genes that determine these signaling properties encode the receptors for specific neurotransmitters or neuromodulators, in addition to neuropeptides and enzymes required for the biosynthesis and transport of small molecule transmitters. Collections of driver lines that use T2A to couple Gal4 expression specifically to genes important in neurotransmission and neuromodulation have recently been made by two laboratories and represent additional important resources for those interested in implementing the Split Gal4 method (Deng et al., 2019; Kondo et al., 2020, Table 1). Both collections consist of lines in which the Gal4 coding sequence is fused to the 3′ end of a native gene encoding a signaling-related molecule *via* the T2A coding sequence. Using vectors made by Kondo et al. (2020), Gal4 can be exchanged for Zip^−^-Gal4DBD and Zip^+^-p65AD by the sequential action of ΦC31 and the recombinase, Cre (Figure 4A, bottom).

#### Converting Enhancer-Trap Gal4 Drivers to Split Gal4 Hemidrivers

Early efforts to map neuronal circuits in the fly by targeted manipulations of activity relied on collections of Gal4 enhancer-trap lines made by P-element transgenesis. Because P-element integration occurs preferentially in the 5’ upstream region of genes in enhancer-rich regions, the expression of Gal4 constructs placed at these sites tends to reflect, albeit imperfectly, the expression of nearby genes (Spradling et al., 1995). Comprehensively characterized Gal4 enhancer-trap collections, such as the NP collection made by Hayashi et al. (2002), which consists of some 4,000 Gal4 lines with 3,825 distinct, mapped genomic insertion sites, thus represented a resource for sampling a wide variety of cell types. Cell groups with desired anatomical or functional properties could be identified in such lines by a variety of methods, including activity manipulations performed with UAS-TNT, UAS-Shi^ts1^, UAS-TrpA1, and other effectors (Gohl et al., 2017; Martin and Alcorta, 2017). In large-scale screens of such lines, the effects of activity manipulations on behavior could be observed and in some cases, the behavioral effects could be mapped to particular neurons (Kohatsu et al., 2011; Flood et al., 2013). Because the expression patterns of most enhancer-trap lines are quite broad, often encompassing many thousands of neurons, additional methods are typically required to restrict the original pattern to smaller subsets of cells.

A general method for converting a Gal4 line with expression in cells of interest into a Split Gal4 line involves the homology assisted CRISPR knock-in (HACK) method developed by Lin and Potter (2016; Figure 4B, top) This method uses a Gal4-specific guide RNA (gRNA) to introduce a Cas 9-mediated double-strand break into the middle of the Gal4 sequence. Donor constructs flanked by Gal4 homologous sequences can then be introduced at the breakpoint by homology-assisted repair. If these constructs are preceded by a T2A sequence, in-frame with the Gal4 sequence at the breakpoint, the new construct will be expressed and translated in addition to a truncated fragment of the original Gal4 molecule. By making transgenic flies bearing the donor construct together with the Gal4 gRNA, the replacement of Gal4 by an alternative construct can be effected *in vivo* by a series of genetic crosses. Flies bearing donor constructs for the Zip^−^-Gal4DBD and Zip^+^-p65AD were introduced by Xie et al. (2018) to permit HACK-mediated conversion of arbitrary Gal4 drivers of interest into Split Gal4 hemidrivers with equivalent expression patterns.

An alternative to converting existing enhancer-trap Gal4 lines into Split Gal4 lines was developed by Gohl et al. (2011) who generated instead a new and large collection of enhancer-trap lines made with a novel Gal4 expression cassette (Table 1). This cassette could be exchanged using ΦC31 and two additional recombinases for any of a variety of alternative cassettes encoding other transcriptional regulators, including Zip^−^-Gal4DBD, Zip^+^-Gal4AD, and Zip^+^-VP16AD (Figure 4B, bottom). Gal4 enhancer-trap lines made using this “integrase swappable *in vivo* targeting element” (inSITE) can be screened similarly to other Gal4 enhancer-trap collections to identify lines of interest, which can then be converted into Split Gal4 hemidrivers with equivalent expression patterns in a straightforward manner.

#### Libraries of Lines Made With Molecularly Defined Cis-Regulatory Modules (CRMs)

Although certain genes are expressed in the nervous system in relatively restricted patterns, most are expressed in many—often many hundreds or thousands of—cells. Co-opting the genetic regulatory elements governing their expression using gene-specific or enhancer-trap methods thus typically results in patterns that are quite broad. To produce Gal4 lines with sparser expression patterns that are more suitable for mapping neural circuits, the laboratory of Gerald Rubin pioneered an alternative strategy (Pfeiffer et al., 2008). Selecting 925 genes expressed in the adult fly brain, they generated 5,200 DNA fragments, each spanning about three kilobases of sequence upstream or downstream of these genes or covering larger introns. These fragments (called cis-regulatory modules, or CRMs) typically contain one or more enhancers, which can be used to drive Gal4 expression when combined with a synthetic promoter. Gal4 driver lines were generated by inserting such constructs into the attP2 landing site on the *Drosophila* 3^rd^ chromosome using ΦC31. The large majority of these drivers showed expression in the nervous system and on average exhibited expression in the central brain in fewer than 100 neurons.

Expanding on the success of this strategy, Jenett et al. (2012) established a collection of 7,000 CRM lines (so-called “GMR” or “Generation 1” lines) in which Gal4 expression was driven by neural enhancer fragments with defined sequence from 1,200 genes. Importantly, these authors also extensively characterized the central nervous system expression of 6,650 of the lines by confocal microscopy and annotated the patterns for anatomical features using machine-assisted methods. A similar effort by the laboratories of Barry Dickson and Alexander Stark at the Institute of Molecular Pathology in Vienna generated some 8,000 Gal4 lines (“Vienna Tiles,” or VT lines) using 7,705 CRMs (Kvon et al., 2014). Initially, characterized by their embryonic expression patterns, a subset of 2,800 lines with restricted expression in the male brain were subsequently imaged using the same methodology described by Jenett et al. (2012). The curated, searchable images of the CNS expression patterns of the GMR and VT lines have been made publicly available *via* the FlyLight Project at the Janelia Farm Research Campus (Table 1).

Because the expression pattern of any GMR or VT line is dictated by the CRM used to express Gal4, the CRM can be repurposed to drive the expression of Split Gal4 components in the same pattern, as long as the Zip^−^-Gal4DBD or Zip^+^-AD construct is introduced back into the same landing site as the original Gal4 construct (Figure 4C). In this manner, Split Gal4 hemidrivers that target the cells lying at the intersection of two overlapping Gal4 expression patterns can be generated. Identifying CRMs that are likely to give an overlapping expression of Split Gal4 hemidrivers in cell types of interest has been greatly facilitated by the development of image registration and analysis tools, such as the color depth “MIP mask” tool (Otsuna et al., 2018), the Neuroanatomy Toolbox (Bates et al., 2020), and the recently released NeuronBridge software (Meissner et al., 2020). Such software tools can be used to align, compare, and search for similar expression patterns from confocal Z-stacks. As illustrated by the examples described in the next section, this procedure has facilitated the selection of suitable CRMs for the production of many Split Gal4 hemidrivers, which have been used in combination to target particular cell types of anatomical or functional interest.

In addition to the many Split Gal4 “drivers” (i.e., specific combinations of hemidrivers that target a cell group of interest) that have been generated in the pursuit of particular biological questions, both the Rubin and Dickson laboratories have produced large libraries of Zip^−^-Gal4DBD and Zip^+^-p65AD lines to serve as building blocks for generating further Split Gal4 pairs of interest (Dionne et al., 2018; Tirian and Dickson, 2017, Table 1). Together, the two groups have made approximately 4,000 Zip^−^-Gal4DBD lines and 3,000 Zip^+^-p65AD lines, which have been deposited at the Bloomington *Drosophila* Stock Center for public distribution. To facilitate genetic pairing of the Split Gal4 components, all Zip^−^-Gal4DBD stocks have transgene insertions on the 3^rd^ chromosome at the attP2 ΦC31 landing site, while all Zip^+^-p65AD stocks have insertions on the 2^nd^ chromosome at attP40. Dionne et al. (2018) describe a pipeline for rationally generating Split Gal4 drivers that target cell types of interest from the lines in these collections. Also, these authors provide useful guidelines and notes of caution. Based on the collective experience of several groups working with the FlyLight lines at the Janelia Research Campus and approximately 20,000 crosses, they note that highly specific intersections that include only the target cells of interest are a rarity occurring in no more than 5% of cases. However, it is not uncommon to generate multiple sparse intersections the only common element of which is the neurons of interest. In this manner, they state that it should be ultimately possible to generate relatively specific lines for three-quarters of the neurons in the adult fly brain using these methods.

## Applications of The Split Gal4 System in Neural Circuit Mapping

In the nervous system, as in all of biology, form and function are tightly coupled. The shapes of different neuronal cell types—where their processes go and what kinds and numbers of contacts they make with other cells—are closely related to the type of information they process and pass on. In facilitating the study of individual cell types, the Split Gal4 method has made critical contributions to studies of both the architecture and operation of the fly nervous system. Indeed, the principal contribution of the Split Gal4 system has been to provide a bridge between the classical disciplines of neuroanatomy and neurophysiology. By enabling the reproducible targeting of the same cell type in different animals, Split Gal4 allows researchers to move seamlessly between analysis of a cell’s connectivity and activity. For some problems, connectivity may provide the most natural entry point—if, for example, one wants to understand what type of information is processed in a particular brain region. In this case, it is important to know which neurons supply input to and carry output from that region, as well as the connectivity of local interneurons. For other problems, a neuron’s anatomy and connectivity may not be of interest initially—as when one wants to understand which neurons govern a particular behavior. In this case, first identifying the functionally relevant neurons is paramount, and piecing together their interactions with each other may be secondary.

The following sections illustrate applications of the Split Gal4 system to problems of both of these types. On the physiological side, the Split Gal4 system has allowed neurons to be targeted so that their activity can be characterized or manipulated in different contexts. Information derived from such experiments is indispensable to understanding whether and how particular neurons contribute to circuit-level function and behavior. On the anatomical side, the Split Gal4 system has facilitated the mapping of dendritic and axonal projections of individual neurons. When done comprehensively for the neuronal types in a particular brain region, this has helped reveal the design principles governing operations of the fly nervous system from motion detection to memory.

### From Anatomy to Function: Split Gal4 in *Drosophila* Systems Neuroscience

Nervous systems are compartmentalized into areas of specialized function that are characterized by the inputs they receive from, and the outputs they send to, other parts of the brain or body. The neurons that receive and send these distinct signals necessarily have morphologies evolved to serve this purpose and defining neuronal cell types according to their morphology and position in the nervous system has been an essential feature of neuroscience research from the time of Cajal. Anatomical methods have enjoyed a renaissance in *Drosophila* since the introduction of the Gal4-UAS system. Recombinase-based methods for stochastically labeling single cells, such as MARCM (Lee and Luo, 1999) and Flp-Out Gal80 (Gordon and Scott, 2009), made it possible to parse Gal4 expression patterns and anatomically catalog the cell types of particular parts of the brain (see for example Jefferis et al., 2007). More recently, the introduction of CRM Gal4 lines and methods such as Flybow (Hadjieconomou et al., 2011), *Drosophila* Brainbow (Hampel et al., 2011), and Multi-color Flp-Out (MCFO; Nern et al., 2015), which permit individual neurons in a pattern to be differentially labeled by distinct fluorescent markers, has further enabled anatomical characterization of specific brain structures (see Wolff et al., 2015). To provide a framework for organizing the emerging knowledge from such studies, a standardized nomenclature for fly neuroanatomy was created in 2014 by Ito et al. (2014).

**Figure 5:**
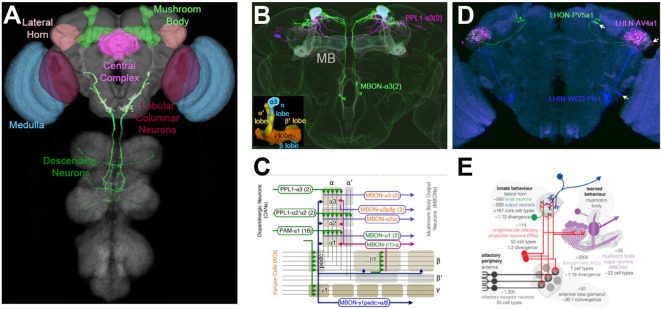
Targeting cell types of anatomical interest. **(A)** Colored regions indicate structures within the *Drosophila* CNS for which comprehensive libraries of cell type-specific Split Gal4 drivers have been made. Targeted cell types include those of the medulla (blue) and lobula (red) in the optic lobes. In the central brain, cell types of the mushroom body (MB; light green), lateral horn (LH; light brown), and parts of the central complex (magenta) have been targeted. Also, over 100 Split Gal4 drivers have been made that target diverse neuron types that send descending projections from the brain into the ventral nerve cord (dark green). See text and Table 1 for references and details. **(B)** Examples of Split Gal4 drivers that target MB neurons. One driver, PPL1-α3(2), labels two dopaminergic input neurons (magenta) with axonal projections to the α-lobe. The MBON-α3(2) driver labels two output neurons (green) with dendritic fields in the α-lobe. The overlapping expression appears light blue and MB lobes are shaded lightly in white. Inset: the five lobes of the MB formed by Kenyon Cell axons. α, α’ lobes, vertical blue and yellow, respectively; β, β’ lobes, horizontal blue and yellow, respectively; γ lobe, orange. The α3 compartments of the α-lobe, which is targeted by both the PPL1-α3 and MBON-α3 processes are outlined. **(C)** The topology of MB circuits deduced from the anatomical analysis of the MB Split Gal4 drivers. The axons of the Kenyon Cells (gray lines) form the lobes of the MB, which divides into 16 compartments (rounded rectangles). Each compartment is characterized by its distinct inputs (mostly dopaminergic PPL and PAM neurons) and outputs (MB output neurons, MBONs), examples of which are shown. **(D)** Examples of Split Gal4 drivers that target local (LHLN; magenta), input (LHIN; blue), and output (LHON; green) neurons of the LH. Driver names are as indicated. Arrows point to cell bodies. **(E)** Schematic summarizing the anatomy and function of LH neuron types and their roles in olfactory processing relative to the MB. Panel **(A)** generated from Virtual Flybrain, with DN from Namiki et al. (2018); panels **(B,C)** adapted from Aso et al. (2014a); panels **(D,E)**, adapted from Dolan et al. (2019).

While anatomical methods may provide essential clues about the functions of individual neurons, they must be supplemented by experimental manipulations to establish what roles a given neuron plays. By permitting first the anatomical, and then the functional, characterization of specific cell types, Split Gal4 targeting methods are allowing just such questions to be answered for diverse parts of the fly brain. Spearheaded largely by the efforts of the Rubin lab at the Janelia Research Campus and their collaborators, several collections of anatomically selective, stable “Split Gal4 drivers” have been created (Figure 5A). These drivers—each of which consists of a particular pair of Zip^−^-Gal4DBD and Zip^+^-AD hemidrivers combined in a single fly—can be used to systematically target cell types innervating parts of the optic lobe (Tuthill et al., 2013; Wu et al., 2016; Davis et al., 2020), the central complex (Wolff and Rubin, 2018), the lateral horn (Dolan et al., 2019; Frechter et al., 2019), and the mushroom body (Aso et al., 2014a,b). Also, a large collection of Split Gal4 drivers has been generated that targets neurons with somata in the brain and descending projections to motor processing regions of the ventral nerve cord (Namiki et al., 2018). Together, these collections have provided key insights into how the fly nervous system processes visual information, forms and express associative memories, exercises and maintains sensorimotor control, and processes innate behavioral responses to odors. Although a detailed description of the landmark articles that introduced each of these collections is well beyond the scope of this review, the nature, and importance of each collection will be briefly discussed, with a particular focus on the collections that cover the lateral horn and mushroom body.

#### Visual System Split Gal4 Drivers: the Optic Lobe

The first collection of cell-type-specific Split Gal4 drivers to be made targeted each of the 12 non-photoreceptor cell types innervating the lamina, the first of four visual neuropils in the optic lobe (Tuthill et al., 2013). This collection differs somewhat from the others described here, in that the anatomy of all but one of the 12 cell types targeted had been well-described by classic Golgi studies (Fischbach and Dittrich, 1989) and electron microscope reconstructions (Meinertzhagen and O’Neil, 1991; Rivera-Alba et al., 2011). However, the individual functions of these cell types in motion detection—a key aspect of visual processing in which the lamina was thought to have a critical role—was largely unknown and activity suppression experiments by Tuthill et al. (2013) using the cell-type-specific drivers established that four of the 12 lamina neuron types contributed to this process. These lines have subsequently been used in over a dozen studies that have refined these original results (Borst et al., 2020).

A major output region of the optic lobe that has been analyzed using Split Gal4 methods is the lobula. Projection neurons (VPNs) from this area convey processed visual information to other parts of the brain and Wu et al. (2016) created a set of VPN-specific Split Gal4 drivers that individually target each of 22 distinct lobula columnar cell types. Over half of these were unknown from previous work. Using the VPN-specific drivers, the authors characterized the response properties of all 22 cell types to visual stimuli, mapped their projections to areas within the central brain, and analyzed the behavioral consequences of their activation. More recently, these neurons—together with the 12 lamina cell types and 42 additional optic lobe neuronal cell types selectively targeted by newly developed Split Gal4 drivers—have been subjected to comprehensive transcriptomics analysis to determine their mechanisms of intercellular signaling (Davis et al., 2020). Analyzing certain cell types known to be synaptically connected from EM reconstructions of the optic lobe, the latter study revealed the previously unrecognized use of acetylcholine, rather than histamine, as a neurotransmitter at certain photoreceptor synapses. In this way, Split Gal4 methods have served not only to define cell types of anatomical interest, but to bridge neuroanatomical detail to neurophysiology, connectomics, and genomics.

#### Split Gal4 Drivers for the Central Complex and Descending Neurons

One important recipient of visual information in the fly brain is a structure consisting of several neuropils collectively called the central complex (CC). The CC contributes to a wide range of behaviors related to the animal’s orientation in space and has recently been shown to form an explicit representation of a fly’s directional “heading” in a structure called the Ellipsoid Body, which receives inputs from neurons in the Protocerebral Bridge (PB; Seelig and Jayaraman, 2015). A comprehensive collection of Split Gal4 drivers targeting neurons of the PB, together with two other CC neuropils, has recently been characterized by Wolff and Rubin (2018) and is complemented by an additional small set of functionally characterized CC Split Gal4 drivers described by Franconville et al. (2018).

The integration of sensory information by structures such as the CC must eventually be conveyed to motor processing areas and translated into behavior. Because behavior requires movements of the legs and wings, which are located on the thorax and are controlled by neurons in the thoracic ganglia of the ventral nerve cord, information must be transferred from areas in the brain to these ganglia. The neurons responsible for this transfer (so-called descending neurons, or DNs, see Figure 5A) typically have their cell bodies and dendritic arbors in the brain and axons that pass through the neck and terminate in the VNC. A comprehensive collection of 133 Split Gal4 drivers targeting 54 distinct types of DNs was made and anatomically characterized by Namiki et al. (2018). In distinction to the other cell-type-specific Split Gal4 collections described above, which typically label multiple morphologically similar cells, the DN collection consists of lines that typically label a single pair of bilateral DNs. The pioneering study describing these lines provided a detailed map of the leg and wing motor neuropils of the thoracic ganglia to which the different DNs project. Also, it described a novel integrative region between these two neuropils that receives input from a broad range of brain areas and may control both sets of appendages. An accompanying study by Cande et al. (2018) characterized the behavioral effects of individually activating the DNs in which these lines express. Their assay was biased against the observation of flying movements but found many neurons that selectively produced walking, tapping, reaching, or grooming phenotypes.

#### Olfactory System Split Gal4 Drivers: Mushroom Body and Lateral Horn

In addition to the systems that govern visual and motor processing in the fly, the olfactory system has also been a major focus of study. The last two Split Gal4 driver collections to be discussed here were created to elucidate the cellular basis of olfactory processing in two distinct brain areas known for their very different handling of odor and pheromone information. Both regions receive input from projection neurons of the antennal lobe, the second-order processing station of the olfactory system, but one region, called the mushroom body (MB), transforms this input into context-dependent memories that permit flexible, experience-dependent responses to odors, while the other, called the lateral horn (LH) appears to encode stereotyped, innate responses to odors. Although the MB had attracted intense interest because of its role in associative learning, the connectivity of its component neurons was only partially characterized until Aso et al. (2014a) created 92 Split Gal4 drivers that comprehensively described the essential MB cell types (Figures 5B,C). Similarly, knowledge of the cellular composition of the LH was fragmentary before two recent Split Gal4-based analyses that enumerated and characterized its neuronal composition (Frechter et al., 2019; Figures 5D,E; Dolan et al., 2019).

The MB consists of three basic cell types: a large number of Kenyon cells (KCs) which receive randomly distributed input from olfactory PNs; MB output neurons (MBONs) which receive synaptic input from the KCs and broadly translate it into the approach or avoidant behaviors; and dopaminergic neurons (DANs), which modulate the KC-MBON synapses. Mutant studies initiated in the laboratory of Martin Heisenberg in the late 1970s had suggested an elegant model of MB connectivity (Heisenberg, 2003) the basic details of which were decisively confirmed and considerably refined by Aso et al. (2014a,b) in two sweeping and insightful studies. These studies bared the basic logic of MB operations by precisely defining the input-output relations of individual DANs and MBONs using Split Gal4 lines (Figure 5C). The MB consists of distinctive “lobes” formed by the KC axons, and the two studies showed that these lobes are parcellated into 15 compartments, each of which is occupied by the dendrites of 1–4 specific MBONs and the synaptic terminals of similar numbers of specific DAN cell types. Because DAN activity, in general, reflects the rewarding or aversive impact of environmental conditions, and because DANs modulate the strength of KC-MBON synapses, rewards and punishments become associated with particular odors by activity within the MB network. The broad influence of this work can be recognized in the fact that the collection of MB Split Gal4 drivers has been used in at least 30 subsequent studies to date, and the basic model of MB network function has attracted attention from a range of researchers including those working at the interface of neuroscience and artificial intelligence (Srinivasan et al., 2018).

Whereas the Split Gal4 investigation of MB circuitry was anticipated by work carried out with Gal4 enhancer-trap lines (Tanaka et al., 2008), the second major processing area for olfactory information, the LH, had proved relatively resistant to such approaches. The Jefferis laboratory, therefore, took a two-pronged approach to mapping and characterizing LH neurons. On the one hand, they generated a large set of Zip^−^-Gal4DBD and Zip^+^-VP16AD enhancer-trap lines, from which they selected 234 hemidriver pairs with distinctive expression in LH neurons that could be used for physiological characterization (Frechter et al., 2019). On the other hand, they generated 210 stable Split Gal4 drivers based on anatomical screening of the GMR and VT library lines that collectively expressed in 82 distinct LH cell types (Dolan et al., 2019). Fifty-three of these cell types were specifically labeled by individual Split Gal4 drivers and included local (LHLN), input (LHIN), and output (LHON) neurons (Figure 5D). Using these two approaches, the authors demonstrated that the LH is considerably more diverse in cell-type composition than the MB. In contrast to MB cell types, individual LH cell types generally displayed stereotyped response profiles to odorants consistent with the genetic—as opposed to experience-dependent—encoding of olfactory information by the LH. Interestingly, LH output neurons, which have long been thought to play an important role in innate behavioral responses to odors and pheromones were found not to project directly to motor processing areas. However, a significant fraction (~30%) had processes that overlapped significantly with those of DANs or MBONs, suggesting that an interplay between innate and learned responses to odors might be critical in interpreting olfactory information (Figure 5E).

### Functional Screens: Split Gal4 Mapping of Neural Circuits Governing Behavior

The use of Split Gal4 methods to study the neural circuits that govern behavior has its roots in the systematic screens initiated by Seymour Benzer’s lab in the 1960s to identify genetic mutants with behavioral deficits. These screens inspired the subsequent cell-based screens conducted using enhancer-trap Gal4 lines mentioned above. Just as genetic screens required the subsequent identification of the actual mutation that caused a behavioral deficit, so enhancer-trap methods required isolation of the actual neurons within a Gal4 pattern that caused a behavior change when blocked. The Split Gal4 method was introduced precisely to permit such refinement of a Gal4 expression pattern and its considerable utility in this regard has been demonstrated in numerous behavioral screens conducted with the collections of GMR and/or VT Gal4 lines. Among these are studies that have successfully identified and/or characterized neural substrates of: grooming (Hampel et al., 2015, 2017), walking (Bidaye et al., 2014; Robie et al., 2017; Sen et al., 2017, 2019), gap-crossing (Triphan et al., 2016), male aggression (Hoopfer et al., 2015; Watanabe et al., 2017; Duistermars et al., 2018; Jung et al., 2020), female mating receptivity (Feng et al., 2014), egg-laying (Shao et al., 2019; Wang et al., 2020), circadian rhythms (Guo et al., 2017; Liang et al., 2019; Sekiguchi et al., 2019) and sleep (Liu et al., 2016). Increasingly, the Split Gal4 method is being integrated into powerful circuit-mapping pipelines that employ high-throughput screening methods in which behavioral analysis is facilitated by machine learning and other computational approaches (Dankert et al., 2009; Anderson and Perona, 2014; Robie et al., 2017; Cande et al., 2018).

#### Split Gal4 Dissection of the Circuit Governing Backward Walking and Crawling

**Figure 6:**
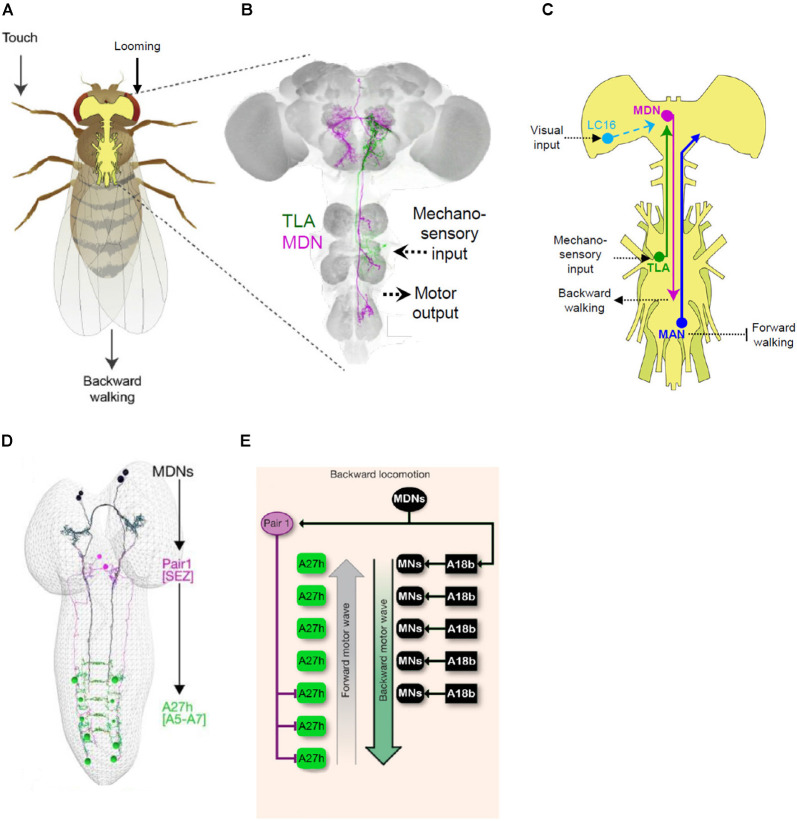
Mapping behavioral circuits using Split Gal4. **(A)** Backwards walking (“moonwalking”) in adult *Drosophila* can be evoked by both mechanosensory and visual stimuli. **(B)** Two distinct neurons identified by Split Gal4 methods mediate reversal of walking direction: “Moonwalker Descending Neurons” (MDN, magenta) and “Two Lumps Walking Ascending Neurons (TLA, green). **(C)** Schematic showing the circuitry governing backward walking deduced from several Split Gal4 studies. In addition to the MDN and TLA, essential neurons in the circuit include lobula columnar (LC16) and mechanosensory neurons (TLA), which are sensitive to looming stimuli and touch, respectively, and the “Moonwalker Ascending Neurons” (MAN), which suppress forward walking. **(D)** Split Gal4 screening to find neurons involved in backward crawling also identified the MDNs (black). Connectomics analysis revealed that a major target of the larval MDNs is a pair of GABAergic neurons in the SEZ, “Pair1” (magenta), which inhibit posterior A27 h premotor neurons (green) required for forward crawling. **(E)** Schematic showing the larval circuitry responsible for backward crawling (i.e., “mooncrawling”). Panels **(A–C)** adapted from Sen et al. (2019); panels **(D,E)** from Carreira-Rosario et al. (2018).

An instructive example of how Split Gal4 is facilitating circuit-mapping studies comes from the study of backward walking in the fly. Flies, like other animals, can respond to obstacles and potential threats by reversing their direction of locomotion. This reversal, however, does not simply invert the sequence of leg movements of the tripod gait normally used for forward walking, but instead invokes less coordinated waves of backward leg movements, first on one side and then the other. How the nervous system generates this novel pattern was unknown until the laboratory of Barry Dickson began investigating it in a series of elegant studies beginning in 2014 (Figures 6A–C). In a behavioral screen of 3,460 VT Gal4 lines, Bidaye et al. (2014) identified four lines that when activated caused flies to walk backward, a phenotype they dubbed “moonwalker.” One line in particular (VT50660), exhibited consistent backward walking when the neurons in which Gal4 was expressed were activated. Conversely, when the activity of these neurons was suppressed, flies failed to reverse direction when confronting a dead end in a linear track.

The VT50660 expression pattern includes seven distinct cell types, two of which were implicated in backward walking by stochastic methods of neuronal activation. Generation of Split Gal4 hemidrivers from VT50660 and several other VT Gal4 lines with expression in these neurons allowed the authors to selectively manipulate each cell type separately. They found that a single pair of neurons with cell bodies in the brain and projections to the ventral nerve cord (“moonwalker descending neurons,” MDN; Figure 6B) was responsible for the moonwalker phenotype, but that the second pair with cell bodies in the VNC and projections to the subesophageal zone (moonwalker ascending neurons, MAN) facilitates backward walking, apparently by inhibiting the program for forward walking. A subsequent high-throughput neuronal silencing screen by the Dickson lab assayed several thousand VT Gal4 and Split Gal4 driver lines for animals impaired in backward walking when confronting a dead end (Sen et al., 2019). Reversal of walking under this condition is thought to depend, in part, on mechanosensitive neurons activated by contact with the barrier and indeed the screen produced one Split Gal4 driver, which the authors named “Two Lumps Walking.” This driver included ascending neurons with arbors in the mesothoracic ganglia and projections that overlapped with those of the MDNs. Anatomical screening of the VT Gal4 collection identified a line with expression in these particular neurons, but not in other neurons present in the original Split Gal4 line. By combining hemidrivers generated from this line with hemidrivers from the original line, the authors were able to selectively label and manipulate the activity of the ascending neurons (Two-lumps Ascending, TLA) and show that they mediated mechanosensitive input to the MDNs to govern reversal of walking (Figures 6B,C).

In an example of how Split Gal4-based circuit mapping approaches can productively synergize, Wu et al. (2016) in their analysis of lobula columnar neurons identified a subset (LC16) that also triggered a moonwalker-like phenotype when activated. Although the LC16 and MDN neurons do not have synaptic contacts, Sen et al. (2017) showed that activation of LC16 neurons is sufficient to activate the MDNs and that silencing of the latter neurons blocks the moonwalking phenotype elicited by stimulation of the LC16 neurons. Because the LC16 neurons are thought to mediate visual responses to looming, these studies collectively indicate that the MDN neurons act as central coordinators of evasive locomotor responses to both visual and mechanosensory input (Figure 6C). Although the manner in which the MDNs act on motor circuits to induce backward walking in adults remains to be characterized, significant progress on this issue has been made in the larva, where the same neurons are present and have been shown to induce a backward crawling (“mooncrawler”) phenotype (Carreira-Rosario et al., 2018).

As in the adult, the observation that the polarity of larval crawling could be reversed by activation of specific neurons came from a screen of CRM Gal4 lines. Split Gal4 refinement produced three different lines with the mooncrawler phenotype that overlapped in expression only in a small complement of bilateral descending neurons. Using the KZip^+^ to eliminate unwanted expression in the VNC and a stochastic labeling strategy to isolate other neurons within the pattern, Carreira-Rosario et al. (2018) were able to show that two pairs of the descending neurons were responsible for the mooncrawler phenotype (Figure 6D). Using the morphological features of the larval MDNs as guides, they were able to identify them in electron micrograph reconstructions of the larval connectome and map their connectivity. Paired activity manipulation/monitoring experiments of the MDNs and distinct subsets of downstream neurons allowed the authors to demonstrate that the MDNs exert two fundamental actions on the locomotor circuit: they directly activate an excitatory premotor neuron important for backward crawling (A18b; Figure 6E) and simultaneously inhibit the forward crawling circuitry *via* disynaptic inhibition of a second excitatory premotor neuron (Figures 6D,E). Although details of MDN connectivity must necessarily differ in the adult—where the motor circuitry is housed in the thoracic, rather than abdominal, ganglia and governs movement of the legs rather than the body wall—the fact that both the larval and adult circuits share an essential “command-like” element (i.e., MDN) suggests that common principles apply to the governance of backward locomotion at both developmental stages. The identification of this element in addition to major sensory inputs and motor outputs within the space of 5 years is also testimony to the power that Split Gal4 methods lend to modern strategies for circuit-mapping in the fly.

### Split Gal4 Synergies With Connectomics: Larval Neural Circuits

Recent progress in single-cell transcriptomics and electron microscopy (EM) is defining cells of the nervous system with unprecedented granularity. As these methods permit the discrimination of ever more refined categories of neurons based on their patterns of gene expression or their connectivity, the Split Gal4 method has assumed increasing importance as a way to examine the function of new types of neurons. The investigation of functionally interesting neurons discovered using Split Gal4 has conversely benefited from the consummate neuroanatomical detail afforded by recent EM reconstructions. Researchers have been able to leverage connectomics data to identify not only the immediate synaptic partners of the neurons they have identified but also other parts of the circuits in which they participate. The value of combining Split Gal4 and EM data was already evident from early studies. Targeted manipulations of neurons downstream of UV-sensitive photoreceptors together with serial-section EM reconstruction of the fly medulla established the Dm8 amacrine neurons as the substrates governing flies’ attraction to UV light (Gao et al., 2008; Meinertzhagen, 2018). However, the explicit interplay of Split Gal4 targeting and connectomics data has more recently been fostered by the ambitious goals of Janelia’s FlyLight and FlyEM projects. These projects aim to produce Split Gal4 reagents for investigating most of the cell types in the fly brain, while also providing a complete map at a synaptic resolution of the entire fly nervous system. The benefits of this combined approach can be seen in work that incorporates data from EM reconstructions of the optic lobe (Shinomiya et al., 2019) and mushroom body alpha lobe (Takemura et al., 2017), as well as the just-completed adult “hemibrain” (Zheng et al., 2018; Wang et al., 2020). Nowhere is the value of bootstrapping EM and Split Gal4 data more evident, however, than in studies of the larval nervous system.

**Figure 7:**
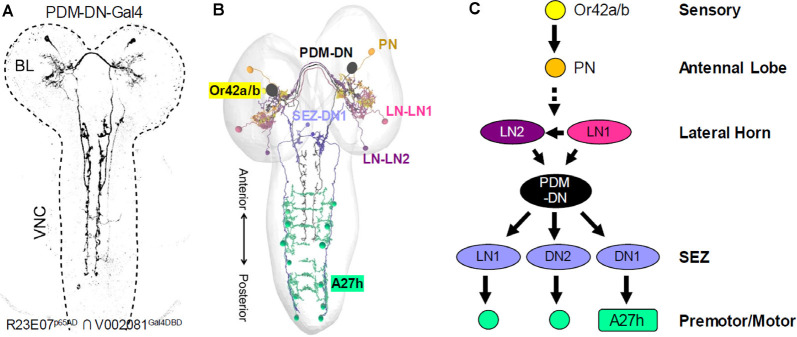
Mapping larval sensorimotor circuits using Split Gal4 and connectomics. **(A)** The R23E07^p65AD^ ∩ V002081^Gal4DBD^ Split Gal4 driver was identified in a synaptic suppression screen to identify neurons involved in larval chemotaxis. This driver labels a single pair of descending neurons (PDM-DN). **(B)** Representation of the EM reconstructed neurons in the circuit for larval chemotaxis. Connectomics analysis revealed that the PDM-DN receives input from two lateral horn neurons, LH-LN1 and LH-LN1 (light and dark purple, respectively) and innervate three neurons in the SEZ, one of which is shown (SEZ-DN1, blue). The LH neurons are downstream of unpaired olfactory projection neurons (PN, orange) that receive input from Or42a and Or42b olfactory receptor neurons (yellow). The SEZ-DN1 neuron is the same SEZ neuron identified downstream of the larval “mooncrawler” neurons (i.e., Pair1) and connects to the posterior A27 h premotor neurons (teal). **(C)** Although certain details remain to be determined, such as the identity of the PNs that innervate the LH neurons and the functional interactions of the LH and PDM-DN neurons, the identified components of the larval chemotaxis circuit span the entire neuraxis from the sensory periphery to the final common pathway of the motor neurons [Adapted from Tastekin et al. (2018)].

The small size and numerical simplicity of the larval nervous system made it an attractive candidate for EM reconstruction, a task which was spearheaded by Albert Cardona’s group and has been carried out in collaboration with a variety of researchers interested in different aspects of larval behavior. As in the adult, a major focus of investigation in the central brain has been the MB. Using 12 specific Split Gal4 drivers, Saumweber et al. (2018) functionally characterized a subset of DANs and MBONs in the 3^rd^ larval instar MB, incorporating anatomical insights drawn from a nearly complete EM reconstruction of this structure in the first larval instar (Eichler et al., 2017). Another class of larval circuits whose investigation has benefited from combined EM and Split Gal4 analysis are the dense sensorimotor networks that regulate forward and backward locomotion (Heckscher et al., 2015; Carreira-Rosario et al., 2018; Kohsaka et al., 2019) as well as responses to mechanosensory stimuli (Ohyama et al., 2015; Jovanic et al., 2016, 2019). Interestingly, a screen of approximately 300 Split Gal4 drivers to identify neurons required for tracking odor plumes (Tastekin et al., 2018) labeled a pair of descending neurons (PDM-DN; Figures 7A,B) with anatomy and connectivity similar to that of the mooncrawler neurons described above (Carreira-Rosario et al., 2018). Connectomics analysis revealed that the PDM-DN neurons synapse downstream on an inhibitory neuron in the SEZ also targeted by the MDNs (“Pair 1” in Figure 6D, SEZ-DN1 in Figures 7B,C), which block forward locomotion. It does so by inhibiting the activity of a specific subset of posterior premotor neurons known as A27 h (Figures 6E, 7B,C), which had been previously implicated in forward peristalsis and shown to connect to known motor neurons (Fushiki et al., 2016). On the upstream side, the PDM-DNs were shown to receive prominent input from two LH neurons (LH-LN1/2; Figures 7B,C). These neurons are downstream of identified olfactory projection neurons that mediate responses to odors detected by known olfactory receptor neurons. Remarkably, this means that the basic connectivity of at least one larval sensorimotor circuit has been characterized by the neurons that mediate an initiating sensation to the motor neurons that mediate part of the behavioral response.

## Other Areas of Application and Spin-Offs

While the Split Gal4 method has been primarily embraced by researchers interested in elucidating neural circuits in the fly brain, its range of potential applications is much broader. Within neuroscience, the areas of neurodevelopment and neuromodulation have both benefited from the application of Split Gal4 to certain problems and the use of the method will likely expand in these areas. Outside of neuroscience lies a largely unexplored domain of application, namely other tissues. With its extreme cellular diversity, the nervous system is the tissue most obviously in need of combinatorial methods for isolating functionally and anatomically distinct cell types, but many other tissues are composed of different cell types that can be only incompletely isolated using binary targeting systems. Other binary systems—and other organisms—have also begun to benefit from adaptations of the Split Gal4 technology. In this section, we briefly review these emergent domains of Split Gal4 implementation.

### Neurodevelopment

**Figure 8:**
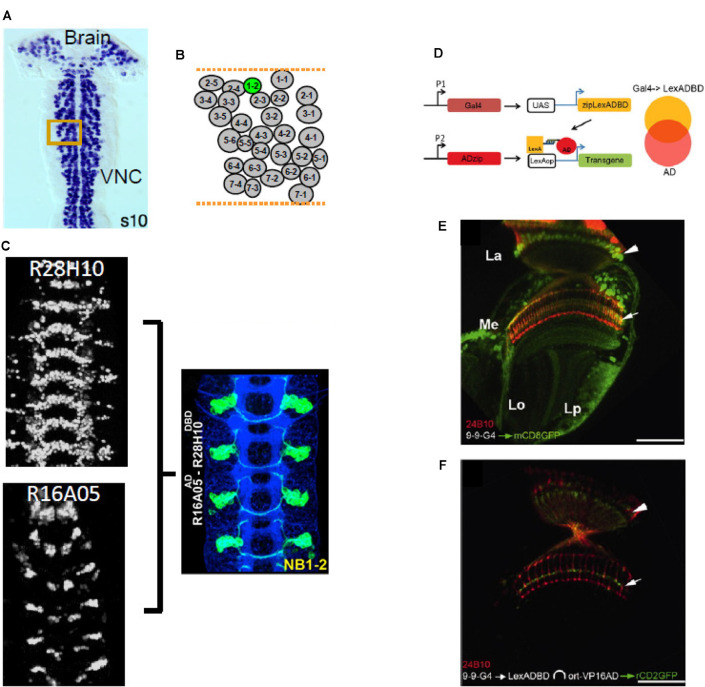
Split Gal4 applications in neurodevelopment and technical spinoffs. **(A–C)** Split Gal4 has been used to target neurons in specific neuronal lineages. **(A)** Larval neurons, some of which persist into adulthood, are born embryonically from neural stem cells called neuroblasts (NBs). Each hemisegment in the ventral nerve cord (VNC), has a similar complement of identified NBs, arranged in rows and columns. **(B)** Diagram of the arrangement of the NBs in a VNC hemisegment, with NB1–2 highlighted in green. **(C)** Two CRM-Gal4 lines (left panels) overlap in expression in NB1–2, as indicated by the Split Gal4 driver made using the two CRMs (right panel). Neuronal progeny of NB1–2 is labeled with GFP (green). **(D–F)** The Split LexA system uses the same heterodimerizing leucine zippers as Split Gal4. **(D)** Using a UAS-Zip^−^-LexADBD, a Gal4 expression pattern can be converted into a Split LexA expression pattern. Where this pattern intersects with the expression pattern of a Zip^+^-VP16AD driven by its own enhancer (P2), a functional LexA::VP16AD transcriptional activator is produced and LexA_op_-mediated transgene expression occurs. **(E)** The 9–9-Gal4 driver expresses in multiple subsets of optic lobe neurons, including the Ort-positive, L3 neurons of the lamina (somata, arrowhead; axon terminals, arrow). **(F)** The L3 neurons can be isolated by using 9–9-Gal4 to drive UAS- Zip^−^-LexADBD in combination with an ort-driven Zip^+^-VP16AD hemidriver. Red, 24B10 immunolabeling. Panel **(A)** from Luan et al. (2020); panel **(C)** adapted from Lacin and Truman (2016); panels **(D–F)** from Ting et al. (2011).

The successive restrictions of cell fate that give rise to neuronal cell types start before neurogenesis and proceed through a series of key developmental events including neurite elaboration and pathfinding, synaptic partner recognition, and sometimes neurite pruning and cell death. To distinguish the cell-autonomous and non-cell-autonomous mechanisms that guide each of these processes, it is often necessary to genetically mark and/or manipulate single cells. Not coincidentally, the first techniques for genetically labeling single cells, such as MARCM (Lee and Luo, 1999), were developed for use in neuroscience. A wide range of powerful genetic techniques for studying neurodevelopment has followed, particularly for use in neuronal lineage mapping (Yu et al., 2009; Awasaki et al., 2014; Ren et al., 2016; Garcia-Marques et al., 2019). Although the availability of these techniques has somewhat mitigated the need for Split Gal4, the latter method has also found productive application in the study of numerous developmental processes. These include neuronal differentiation (Seroka and Doe, 2019), target matching and synaptogenesis (Couton et al., 2015; Courgeon and Desplan, 2019; Menon et al., 2019; Xu et al., 2019), and neuron-glia interactions (Coutinho-Budd et al., 2017; McLaughlin et al., 2019; Shimozono et al., 2019). Also, the characterization of postembryonic neuroblast lineages has profited from the application Split Gal4 methods (Lacin and Truman, 2016; Lacin et al., 2020; Figures 8A–C). Split Gal4 lines generated using CRMs known to express in embryonic neuroblasts (Manning et al., 2012) have been used to permanently label early-born neuronal progeny. While these lines tend to show transient expression of the Split Gal4 components in neuroblast progeny, lines generated using the Trojan exon method and targeting transcription factor genes important for specifying neuronal identity have the advantage of exhibiting persistent patterns of expression of the Zip^−^-Gal4DBD and Zip^+^-p65AD components (Lacin et al., 2019).

### Neuromodulation

Although synaptic signaling between neurons is of paramount importance, neurons also communicate through other channels. One of the most important of these uses not fast neurotransmitters, which directly regulate ionic conductances, but instead, molecules that act on slower timescales—often *via* G-protein coupled receptors—and over larger distances. These molecules, which include an assortment of factors from biogenic amines to neuropeptides, act to modulate synaptic signaling and are called neuromodulators. Specific neuromodulators play important roles in specifying behavioral and physiological states. Identifying the sources of these factors and their sites of action is therefore important to understanding the nervous system function. Mapping such patterns of neuromodulatory connectivity requires selectively targeting neurons that express specific neuromodulators or their receptors. Although Split Gal4 methods offer considerable promise in this endeavor, they have been used only in a small number of cases thus far.

One area where progress is most evident is in the study of molting. This developmental process is particularly reliant on the use of neuromodulators to control behavioral and physiological events (White and Ewer, 2014). Three hormones involved in this process, all of which act within the CNS as neuromodulators, are Ecdysis Triggering Hormone (ETH), Bursicon, and Crustacean Cardioactive Peptide (CCAP). Two of the first applications of Split Gal4 technology—both described above—were used to identify subsets of neurons that released CCAP (Luan et al., 2006) and Bursicon (Luan et al., 2012). More recently, neurons targeted by ETH, CCAP, and Bursicon have been categorized using Split Gal4 into subsets according to their use of different fast neurotransmitters (Diao et al., 2016, 2017). The use of the Trojan exon method has considerably facilitated these efforts by permitting neurons that express the relevant hormone receptors to be selectively targeted by expression of Split Gal4 constructs. Further progress in mapping what might be called the “neuromodulatory connectome” should be facilitated by the libraries of lines described above that systematically target neurons expressing genes important for neuromodulatory signaling (Deng et al., 2019; Kondo et al., 2020, Table 1).

### Targeting Cell Types in Non-neural Tissues

Much of the excitement surrounding the introduction of the Gal4-UAS method centered around its promise for studying the development of a wide variety of tissues. The 220 enhancer-trap lines generated by Brand and Perrimon (1993) expressed Gal4 in a wide range of embryonic cell types. Although the specificity of expression of these and subsequent Gal4 lines is sufficient to characterize different kinds of cells in many tissues, expression in a single cell type in a single tissue is often not possible because of the pleiotropic expression of most genes. However, combinatorial methods such as Split Gal4 largely remain to be exploited to achieve greater selectivity of expression. Emerging transcriptomics data for a wide range of tissues should make it possible to use the Split Gal4 toolbox to rationally generate lines that target particular cell types based on their expression of distinct genes. An alternative approach is to leverage the large numbers of GMR and VT lines to make Split Gal4 stocks for this purpose. Although many of the CRMs used to create these lines were selected based on their proximity to neuronally expressed genes, many such genes also express outside of the nervous system. The VT lines clearly express in diverse tissue types developmentally (Kvon et al., 2014), and a survey of the GMR lines shows that approximately one-fifth exhibit expression in imaginal discs, which give rise to adult appendages, sensory organs, and reproductive tissues (Jory et al., 2012). Thus, it is likely that these collections, and the Split Gal4 collections currently being generated from them, represent a valuable resource for targeting non-neural tissues.

### Split Gal4 Spin-offs

Beyond specific applications, the Split Gal4 technology has also influenced the development of similar technologies for use in the fly and other genetic model organisms. Two similar split transcription factor systems—both using the same leucine zipper pair used in the Split Gal4 system—have been developed in *Drosophila*. These systems can be used to achieve refined expression of reporters or effectors under the control of either split LexA (Ting et al., 2011) or split QF (Riabinina et al., 2019) transcriptional activators. Both can be used in conjunction with the Gal4-UAS system to simultaneously express different reporters/effectors in two distinct cell groups (Takemura et al., 2011). Ting et al. (2011) also introduced a clever method for converting a Gal4 driver into a Split LexA hemidriver by making flies in which the Zip^−^-LexADBD transgene is placed downstream of the UAS (Figures 8D–F). In addition to these fly-based spin-offs, a ternary expression system based on the zebrafish optimized version of Gal, called “Split KalTA4,” has been shown to work in *D. rerio* (Almeida and Lyons, 2015) and a split QF system that uses an alternative pair of zippers has been demonstrated in *C. elegans* (Wei et al., 2012). In general, these systems have yet to gain the same traction as the Split Gal4 system.

## Conclusion

As the examples above make clear, Crick’s dream of being able to manipulate the activity of specific cell types in the brain has been realized in the fly. Enabled by Split Gal4 methods, such manipulations are defining the functions of a growing number of neurons. Coupled with the knowledge of how these neurons interact, which is rapidly becoming available from EM reconstructions of the larval and adult central nervous systems, Split Gal4 is yielding an increasingly comprehensive picture of the fly brain and how it operates.

In some ways, the success of the Split Gal4 method is remarkable. It implies that many cell types in the fly can be uniquely specified by the activity of only two enhancer domains. A critical question is whether this will prove true of the many neuronal cell types that remain to be characterized. It is worth noting in this regard that existing collections of Split Gal4 drivers, such as those for descending or lateral horn neurons, include only about one-third of the estimated cell types in their respective categories (Namiki et al., 2018; Dolan et al., 2019). Also, some current cell types defined by Split Gal4 line expression, such as the lobula columnar neurons and subclasses of MB Kenyon cells, include hundreds of morphologically similar neurons, which may yet yield to a further subdivision based on more subtle genetic and functional differences. The question of whether Split Gal4 technology will allow all neuronal cell types to be individually targeted is thus likely to hinge not only on technical issues but also on how stringent a definition of cell type one adopts. Nevertheless, there is a reason for optimism. First, the Janelia Research Campus, which has both underwritten and driven much of the recent technical progress in fly neuroscience, is continuing to generate further lines and has projected that current methods should allow Split Gal4 combinations to be made that cover 75% of all cell types in the adult brain. The coverage of neurons in the numerically simpler larval brain is likely to be better. Resources created to exploit the many thousands of enhancers represented in the GMR and VT collections will help distribute this effort (see for example Meissner et al., 2020), and methods for rationally identifying novel gene enhancers—or for making gene-specific Split Gal4 hemidrivers—may help realize a relatively complete catalog of Split Gal4 drivers. Where gaps persist and further specificity is required, further restriction using the Killer Zipper or other combinatorial strategies may also help (for examples see Pankova and Borst, 2017; Tison et al., 2019).

A more prosaic question is whether the burden of maintaining many thousands of Split Gal4 lines will represent an impediment to future progress. For stock centers reliant largely on user fees, it is expensive to maintain lines that are infrequently requested, as will generally be the case for cell-type-specific lines. A felicitous feature of the hemidriver lines generated using CRMs from the GMR or VT collections is that they can be regenerated by straightforward means and do not necessarily have to be maintained. The same is true of lines generated using Trojan exons, CRIMIC constructs, or similar methods using 2A peptides. Nevertheless, the cost and effort of remaking lines make alternative methods for sparse targeting of cells attractive, especially if they require maintenance of fewer lines. To date, no other methods have emerged that meet this requirement. The recently developed SpaRCLIn method has been proposed as an alternative to Split Gal4, but its efficacy and promise remain to be demonstrated (Luan et al., 2020).

An obvious lacuna in the Split Gal4 toolbox is the absence of a method for temporally—as well as spatially—restricting transcriptional activity. The standard method of constraining Gal4 activity to a particular time-window using the temperature-sensitive mutant of Gal80 cannot be used with current implementations of Split Gal4 as Gal80 does not bind dVP16AD or p65AD. Possibly the temporal control could be introduced into the Split Gal4 system using dimerization domains that make the association of the Gal4DBD and AD contingent upon light or a chemical inducer of dimerization (Taslimi et al., 2016; Huynh et al., 2020), but these solutions would require the creation of completely new lines. A more congenial solution would be to temporally control Split Gal4 activity by rendering expression (or activity) of the Killer Zipper contingent upon heat or drug binding, perhaps *via* a recombinase, but this has not yet been accomplished.

With these caveats aside, Split Gal4 methods are providing the means for remarkable advances in fly neurobiology. By providing reliable and reproducible genetic access to ever more neuronal cell types, Split Gal4 is enabling the assembly of a comprehensive parts list of the *Drosophila* brain, complete with information about the functions and interactions of these parts. The cornucopia of Split Gal4 lines already available and currently in production can be expected to keep fly neuroscientists busy for some time to come, and as the catalog of lines increases, we can only anticipate a deeper understanding of not only how the fly brain works, but how nervous systems in general help animals navigate the opportunities and risks of the world to promote survival and reproduction.

## Author Contributions

HL and FD: literature search. HL, FD, RS, and BW: figures and writing first draft. BW: writing final draft. RS: collecting copyright permissions. All authors contributed to the article and approved the submitted version.

## Conflict of Interest

The authors declare that the research was conducted in the absence of any commercial or financial relationships that could be construed as a potential conflict of interest.
